# Immune-Enhancing Effects of Co-treatment With *Kalopanax pictus* Nakai Bark and *Nelumbo nucifera* Gaertner Leaf Extract in a Cyclophosphamide-Induced Immunosuppressed Rat Model

**DOI:** 10.3389/fnut.2022.898417

**Published:** 2022-05-19

**Authors:** Young Mi Park, Hak Yong Lee, Dong Yeop Shin, Dae Sung Kim, Jin Joo Yoo, Hye Jeong Yang, Min Jung Kim, Jun Sang Bae

**Affiliations:** ^1^INVIVO Co., Ltd., Nonsan-si, South Korea; ^2^Central Research and Development, Hanpoong Pharm & Foods Co., Ltd., Wanju-gun, South Korea; ^3^Korea Food Research Institute, Wanju-gun, South Korea; ^4^Department of Pathology, College of Korean Medicine, Wonkwang University, Iksan, South Korea

**Keywords:** *Kalopanax pictus* Nakai Bark, *Nelumbo nucifera* Gaertner, immune enhancement, cyclophosphamide, macrophage, immunosuppressed rat, ERK, NF-κB

## Abstract

**Objective:**

Immune system disorders can result in various pathological conditions, such as infections and cancer. Identifying therapies that enhance the immune response might be crucial for immunocompromised individuals. Therefore, we assessed the immune-enhancing effect of co-treatment with *Kalopanax pictus* Nakai Bark and *Nelumbo nucifera* Gaertner leaf extract (KPNN) in a cyclophosphamide (Cy)-induced immunosuppressed rat model.

**Materials and Methods:**

For *in vitro* studies, macrophages and splenocytes were treated with various KPNN doses in the presence or absence of Cy. Macrophage viability, nitric oxide production, splenocyte viability, cytokine production and natural killer (NK) cell activity were analyzed. For *in vivo* studies, analysis of weekly body weight, dietary intake, tissue weight, immune-related blood cell count, cytokine levels, and spleen biopsy was performed in a Cy-induced immunocompromised animal model.

**Results:**

KPNN significantly increased phospho-NF-κB and phospho-ERK protein levels and cell viability in macrophages. KPNN significantly increased the NK cell activity in splenocytes compared to that in the control. Cy treatment decreased tumor necrosis factor (TNF)-α, interleukin (IL)-6, and interferon-γ production. In the Cy-induced immunosuppression rat model, KPNN-treated rats had significantly higher body weights and tissue weights than the Cy-treated rats. Additionally, KPNN treatment restored the immune-related factors, such as total leukocyte, lymphocyte, and intermediate cell contents, to their normal levels in the blood. The blood cytokines (TNF-α and IL-6) were increased, and spleen tissue damage was significantly alleviated.

**Conclusions:**

Collectively, KPNN exerts an immune-enhancing effect suggesting their potential as an immunostimulatory agent or functional food.

## Introduction

An underactive immune system, caused by conditions such as immunodeficiency, is more prone to infection by pathogenic organisms. Thus, immune system disorders can provoke the development of various pathological conditions, such as infections and cancers (1–3). Therefore, identifying therapies that enhance the immune response are vital for immunocompromised individuals.

The immune monitoring function in humans plays a vital role as a biological host defense mechanism against the development of cancer and the proliferation of tumor cells. This anti-tumor immune function is carried out by lymphocytes, macrophages, and natural killer cells (4, 5). The activation of macrophages, B and T lymphocytes are essential steps in the initial regulation of the immune system, which is the defense mechanism of the body (6). Recently, immunotherapy has been used to induce cancer tissue destruction through the activation of cancer antigen-specific T helper (Th) and cytotoxic T (Tc) cells, macrophages, and natural killer (NK) cells (6, 7). The continued activation of immune cells results in the secretion of various cytokines during cell proliferation and differentiation, enabling the continuous amplification and control of immune functions. Therefore, recent studies have focused on discovering natural products and their immune-modulating ingredients that may serve as potential immune boosters, which can be used as ingredients in functional foods (1, 8).

Cyclophosphamide (Cy) exerts anti-tumor and immunomodulatory effects through immunosuppression, myelosuppression, and cytotoxicity (9, 10). Cy can cause a drastic change in the Th1/Th2 ratio bias, resulting in immunosuppression. The immunological effect of Cy reduced lymphocyte proliferation, including that of T cells, which was confirmed by a decrease in the levels of cytokines (tumor necrosis factor (TNF)-α, interferon (IFN)-γ, interleukin (IL)-2, and IL-12) produced by Th1 cells and cytokines (IL-4, IL-6, and IL-10) produced by Th2 cells (11, 12).

Since plants have beneficial pharmacological effects, low toxicity, and fewer side effects on human health, they have traditionally been used in oriental medicine. *Kalopanax pictus* Nakai Bark (KP) and *Nelumbo nucifera* Gaertner leaf (NN) have been shown to have various pharmacological activities, including anti-inflammatory (13–16), anti-nociceptive (14), anti-diabetic (15, 16), and anti-cancer (15–19) effects. The main components of KP and NN have been identified as liriodendrin and kalopanaxaponin (20, 21), and flavonoids and alkaloids (22, 23), respectively. These components are pharmacologically active in KP and NN, and are directly involved in therapeutic efficacy. Natural products are often blended to maximize the therapeutic effect. Although the anti-inflammatory effects of KP and NN extracts have been demonstrated *in vitro* and *in vivo* (13–16), the effects of KP and NN combination (KPNN) treatment on the immune response are yet to be elucidated. Therefore, we investigated the immune-enhancing effects and synergistic effect of KPNN in macrophages, splenocytes, and an immunosuppressed rat model.

## Materials and Methods

### Preparation of Samples

Plant extracts were prepared and purified by Hanpoong Pharm & Foods Co., Ltd. (Jeonju, Jeonbuk, South Korea). Briefly, KP was extracted twice in 50% ethanol (v/v, HPS-3C) at 84–90°C or distilled water (HPS-3A) for 3 h. NN was extracted with 50% ethanol (v/v, HPS-3D) at 84–90°C or distilled water (HPS-3B) for 3 h. Each sample solution was separated through a 5-μm membrane filter, evaporated under reduced pressure at 60°C, and then vacuum-dried. The extract was lyophilized to produce a powder, and aliquots were stored at –80°C until use. KP and NN sample was extracted in water and 50% ethanol, respectively; HPS-3A: KP water extract, HPS-3C: KP ethanol extract, HPS-3B: NN water extract, HPS-3D: NN ethanol extract. The KPNN sample was extracted by mixing KP and NN in 50% ethanol at a ratio of 0.5:1 (KP:NN, HPS-3E), 1:1 (HPS-3F), 1:0.5 (HPS-3G). A screening test was performed using KP, NN, and KPNN extract to determine the optimal concentration and sample showing immune-enhancing effects. Cell viability analysis revealed that RAW 264.7 macrophages and splenocytes had the highest non-toxic concentrations of ≤ 500 and 100 μg/ml, respectively, in all samples (Supplementary Figures 1, 2). Therefore, subsequent *in vitro* experiments were performed at a concentration of ≤ 500 μg/ml. Cy-treated splenocytes viability was analyzed after treatment with the highest non-toxic concentration of the sample or Cy. As a result, HPS-3G (KP:NN = 1:0.5) sample showed the highest cell viability by Cy treatment. When considering cytotoxicity tests and cell viability tests of Cy-induced splenocytes were collected, HPS-3G samples were selected for this study (Supplementary Figure 3). For the animal study, 50, 100, and 200 mg of the tested KPNN (HPS-3G) extract/kg body weight was used. *Escherichia coli* lipopolysaccharide (LPS) and Cy were purchased from Sigma-Aldrich (St. Louis, MO, United States), and HemoHIM was purchased from Kolmar BNH Co., Ltd., (Sejong, South Korea).

### Cell Culture

RAW 264.7 macrophage cell lines were purchased from the Korean Cell Line Bank (Seoul, South Korea) and grown in DMEM (Invitrogen, Carlsbad, CA, United States) culture medium supplemented with 10% fetal bovine serum (FBS) (Gibco BRL, Gaithersburg, MD, United States) with 1% penicillin and streptomycin (Invitrogen) at 37°C in a humidified incubator with 5% CO_2_. Splenocytes were obtained by aseptically dissecting the Wistar rat spleens. The spleen was gently pressed with forceps and passed through a 70-μm cell strainer (SPL Life Sciences, Pocheon, Gyeonggi, South Korea). The cells were harvested and washed thrice in RPMI-1640 (Invitrogen) by centrifugation (80 × *g*, 3 min, 4°C). The cells were then treated with red blood cell lysis buffer (Sigma-Aldrich). Isolated splenocytes were maintained in RPMI-1640 media supplemented with 10% FBS (Gibco BRL) and 1% penicillin/streptomycin (Invitrogen) at 37°C in a humidified incubator with 5% CO_2_.

### Cell Viability Assay

For cell viability analysis, RAW 264.7 cells were seeded in 96-well plates (1 × 10^4^ cells/90 μl/well) and treated with varying concentrations of KPNN or LPS (300 ng/ml) and incubated at 37°C and 5% CO_2_ for 24 h. Additionally, to analyze the viability of Cy-treated splenocytes, the isolated splenocytes were aliquoted at 1 × 10^6^ cells/90 μl/well in a 96-well plate. After incubation for 24 h, various KPNN concentrations were applied to the cells in the absence or presence of Cy (1.6 mg/ml). After incubation for 24 h, the WST-1 (10 μl; ITSBio, Inc., Seoul, South Korea) solution was added to the cell culture medium (100 μl) and incubated for 1 h. The absorbance values were then measured at 405 nm using a Multi-Detection Reader (Infinite 200, TECAN Group Ltd., Switzerland). The percentage of cell viability was calculated using the following equation: (mean OD of treated cells/mean OD of control cells) × 100.

### Nitric Oxide Measurement

Nitric oxide (NO) production was determined as previously described (24). Briefly, after dispensing RAW 264.7 cells in a 48-well plate at a concentration of 8 × 10^4^ cells/400 μl/well, KPNN (0, 30, 50, 100, 300 500, or 1,000 μg/ml) or LPS (300 ng/ml) was added and incubated for 24 h. The supernatant (100 μl) was transferred to another plate. Griess reagent (100 μl) was added, and the plate was incubated at room temperature for 5 min. The absorbance was measured using a microplate reader (TECAN Group Ltd.) at a wavelength of 540 nm. Absorbance was calculated and analyzed according to the standard curve of sodium nitrite.

### Western Blotting Analysis

After dispensing the RAW 264.7 cells (1 × 10^6^ cells/ml) in a 100 mm dish, the samples were treated and incubated for 24 h. Cells were then washed with ice-cold PBS and lysed with lysis buffer (PRO-PREP™ protein extraction solution, iNtRON, South Korea). After disrupting the cell line by vortexing, the lysates were precipitated at 14000 rpm for 10 min at 4°C using a centrifuge. The supernatant was quantified with the same amount of protein lysates using Bradford reagent (Bio-Rad, Hercules, CA, United States). Then, electrophoresis was conducted on a sodium dodecyl sulfate-polyacrylamide gel electrophoresis (SDS-PAGE) gel. The protein was then transferred to a polyvinylidene fluoride (PVDF) membrane and blocked with 5% skim milk solution for 1 h. The membranes were then incubated with primary antibodies overnight at 4°C. After washing the membranes with Tris-buffered saline containing Tween 20 (TBS-T), they were treated with secondary antibodies containing horseradish peroxidase (HRP) for 1 h and then washed with PBS-T. The washed membrane was treated with enhanced chemiluminescence (ECL) solution (EZ-Western Lumi Pico, DoGen, South Korea) and detected using a C-Digit western scanner (LI-COR, Lincoln, NE, United States). The following primary antibodies purchased from Cell Signaling Technology (Beverly, MA, United States) were used: anti-NF-kB p65, anti-phospho-NF-κB p65, anti-p44/42 MAPK (Erk1/2), anti-Phospho-p44/42 MAPK (Erk1/2) (Thr202/Tyr204). Anti-β-actin was purchased from Sigma-Aldrich.

### Natural Killer Cell Activity Assay

Briefly, AR42J rat pancreatic tumor cells obtained from the American Type Culture Collection (ATCC, Manassas, VA, United States) were used as target cells for the NK cell activity assay. Control or KPNN-treated splenocytes were used as effector cells. Splenocytes were co-cultured with AR42J cells in 96-well plates at a 20:1 ratio of effector to target cells and incubated for 24 h. The viability of AR42J cells was assessed using the CytoTox detection kit (TaKaRa, Shiga, Japan) on a microplate reader. NK cell activity was calculated as the viability of AR42J cells compared to that of control cells.

### Cytokine Measurement in Splenocytes

To analyze immune-related cytokine levels, the culture medium was treated with Cy (1.6 mg/ml) and KPNN (5, 10, 30, 50, 100 μg/ml) for 24 h. TNF-α, IL-6, and IFN-γ levels were detected using the Cytokine Activation Analysis Kits (R&D Systems Inc., Minneapolis, MN, United States), according to the manufacturer’s instructions.

### Animals and Experimental Design

Five-week-old specific pathogen-free (SPF) male Wistar rats (*n* = 60) were purchased from Orient Bio Inc., (Seongnam, Gyeonggi-do, South Korea) and adapted to the following conditions (12-h light/dark cycle; temperature, 22 ± 3°C; humidity, 50 ± 5%) for 7 days. The experiment was conducted using a standard diet and drinking water. The experiments were conducted after a week of acclimatization. A model for inducing immunosuppression was established by orally administering Cy (5 mg/kg). The 60 rats were divided into six groups of 10 animals each, after weighing each group: normal control group (normal), immunosuppression group (control), immunosuppression + KPNN 50 mg/kg group (KPNN 50), immunosuppression + KPNN 100 mg/kg group (KPNN 100), immunosuppression + 200 mg/kg group (KPNN 200), and immunosuppression + HemoHIM 1,000 mg/kg group (HemoHIM 1000). All drugs and vehicles were administered orally for 4 weeks. Body weight was measured once per week for clinical evaluation. All animal experiments were approved by the Institutional Animal Care and Use Committee of INVIVO Co., Ltd. (IV-RB-17-2105-11-01).

### Complete Blood Cell (CBC) Count and Cytokine Analyses in Animal Models

Briefly, Wistar rats were orally administered KPNN (0, 50, 100, or 200 mg/kg/day) and Cy (5 mg/kg once per day) for 4 weeks. After the final administration of KPNN, whole blood was collected from the abdominal vena cava of the animals after inhalation anesthesia and divided into ethylenediaminetetraacetic acid (EDTA)-coated tubes (DB Caribe, Ltd., United States) and conical tubes for analysis. The number of total white blood cells (WBCs), lymphocytes, granulocytes, and the mid-range absolute counts (Mid) collected in EDTA-coated tubes were analyzed using a Hemavet 950 counter (Drew Scientific Group, Dallas, TX, United States). For cytokine analysis, blood collected in a conical tube was coagulated at room temperature for 30 min and then separated in a centrifuge at 3000 rpm for 10 min to collect serum. The separated serum was analyzed for TNF-α, IL-6, and IFN-γ levels using an ELISA kit (R&D Systems).

### Histological Analysis

After the animals were euthanized, the spleens were removed, weighed, and the extracted spleen tissue was cut, fixed in 10% formalin solution (trimming), fixed again with 10% formalin solution, embedded in paraffin, and cut into sections with a thickness of 4–7 μm. For hematoxylin-eosin staining, paraffin was removed from xylene and dehydrated, followed by staining with hematoxylin for 4 min and eosin for 2 min. The stained tissue preparations were observed and photographed using an optical microscope (BX50 F4; Olympus, Fukuoka, Japan).

### Statistical Analysis

All experimental results were calculated as mean ± SEM using a statistical analysis program (SPSS ver.12.0, SPSS Inc., Chicago, IL, United States). Statistical analysis to evaluate the significant difference between each experimental group was performed using ANOVA (one-way analysis of variance test) and Duncan’s multiple range tests. Each value represents the mean of at least three independent experiments for each group. Statistical significance was set at *p* < 0.05.

## Results

### Effects of KPNN-Mediated Cell Proliferation and Nitric Oxide Production in RAW 264.7 Cells

**FIGURE 1:**
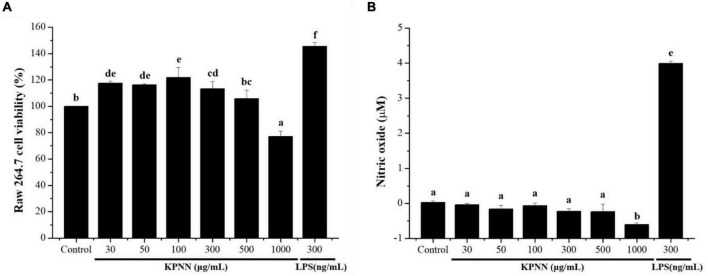
Effect of the KPNN extract on cell survival rate and NO production by macrophages. **(A)** RAW 264.7 cells were seeded in 96-well plates (1 × 10^4^ cells/90 μl/well) and treated with KPNN (0, 30, 50, 100, 300, 500, and 1,000 μg/ml) or LPS (300 ng/ml) and incubated at 37°C and 5% CO_2_ for 24 h. Next, cell viability was measured using a WST-1 assay. **(B)** RAW 264.7, cells were dispensed in a 48-well plate (8 × 10^4^ cells/400 μl/well) and treated with KPNN (0, 30, 50, 100, 300, 500, and 1,000 μg/ml) or LPS (300 ng/ml) for 24 h. NO concentrations in the culture supernatants were assessed using the Griess assay. Bars labeled with different superscripts are significantly different (*p* < 0.05 vs. control). The results are expressed as mean ± SEM of at least three independent experiments (*n* = 3).

To confirm the immune-enhancing effect of KPNN (HPS-3G) extracts on macrophages, toxic concentrations were first tested. The cell viability of the group treated with different KPNN concentrations was analyzed based on the control group to confirm the KPNN concentration that was toxic for macrophages. The viability of RAW 264.7 cells increased in the range of 30–300 μg/ml but decreased in a concentration-dependent manner from a concentration of 500 μg/ml or higher (Figure 1A). An experiment was conducted to analyze the amount of NO production in KPNN-treated RAW 264.7 cells. NO production was not notably different between the KPNN-treated and control groups but was increased in the LPS-treated group (Figure 1B).

### Activation of ERK and NF-κB Proteins by KPNN in RAW 264.7 Cells

**FIGURE 2:**
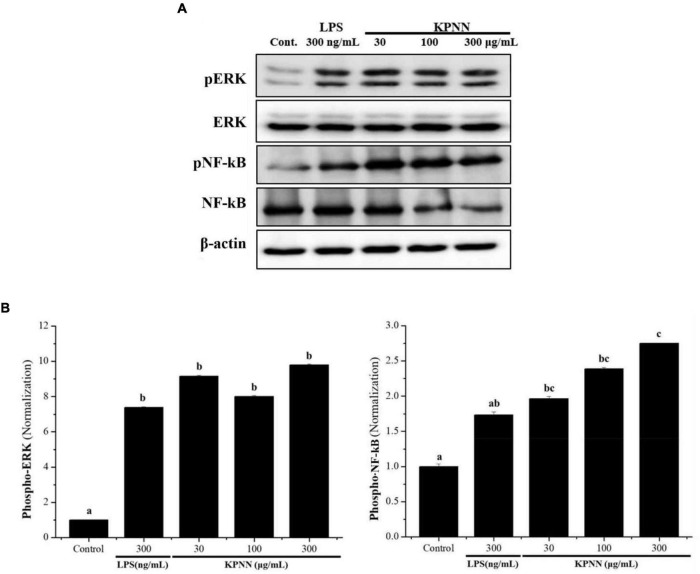
Activation of ERK and NF-κB by KPNN in RAW 264.7 cells. **(A)** RAW 264.7 were aliquoted at 1 × 10^6^ cells/ml in a 100 mm dish/well and treated with KPNN (0, 30, 100, 300 μg/ml) or LPS (300 ng/ml) and incubated for 24 h. The expression levels of phospho-ERK and phospho-NF-κB were examined by western blot analysis using anti-NF-κB p65, p-NF-κB p65, p44/42 MAPK (Erk1/2), and p-p44/42 MAPK (Erk1/2) antibodies. **(B)** Western blots were quantified using the Quantity One 4.6.6 software. Values are presented as mean ± SEM (*n* = 3). Bars labeled with different superscript numerals indicate *p* < 0.05.

To investigate the mechanism of KPNN-mediated immune enhancement, activation of the extracellular-signal-regulated kinase (ERK) and nuclear factor-kappa B (NF-κB) pathways in response to KPNN was evaluated in RAW 264.7 cells by western blotting analysis. Our results showed that KPNN increased the phospho-NF-κB and phospho-ERK levels (Figure 2A). The expression of each protein was normalized, and the relative expression levels of the control group were analyzed. We confirmed that the p-ERK and p-NF-κB protein levels in the KPNN-treated group were higher than that of the LPS (300 ng/ml)-treated (positive control) group (Figure 2B).

### Cell Viability Effect of KPNN on Cy-Induced Splenocytes

**FIGURE 3:**
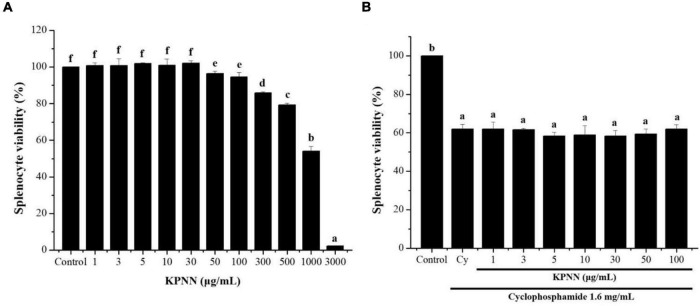
Effects of KPNN on Cy-induced splenocytes. **(A)** Isolated splenocytes were seeded in 96-well plates (1 × 10^6^ cells/90 μl/well) and treated with KPNN (0, 1, 3, 5, 10, 30, 50, 100, 300, 500, 1,000, and 3,000 μg/ml) for 24 h. Then, cell proliferation was determined using a WST-1 assay. **(B)** Splenocytes were seeded into 96-well plates, followed by treatment with KPNN (0, 1, 3, 5, 10, 30, 50, and 100 μg/ml) and Cy (1.6 mg/ml) for 24 h. Cell viability was measured using a WST-1 kit. Data are expressed as mean ± SEM of three independent experiments. *p* < 0.05 vs. control group (*n* = 3). Bars labeled with different superscript numerals indicate *p* < 0.05.

To confirm the cytotoxicity of KPNN against splenocytes, cells were incubated with various KPNN concentrations for 24 h. Cell viability was unaffected at KPNN concentrations of < 100 μg/ml (Figure 3A). Based on these results, 100 μg/ml was set as the optimal non-cytotoxic concentration, and cell viability was analyzed according to Cy (1.6 mg/ml) treatment at < 100 μg/ml KPNN. The analysis of splenocyte viability after treatment with KPNN or Cy revealed that the group treated with Cy alone showed a significant decrease in cell viability to 62.0 ± 2.4% compared to that of the untreated control group. However, the cell viability of the group co-treated with Cy and KPNN was 58–62% at all concentrations, similar to that of the group treated with Cy alone, and no significant difference was observed (Figure 3B).

### Effects of KPNN on Natural Killer Cell Activity in Splenocytes

**FIGURE 4:**
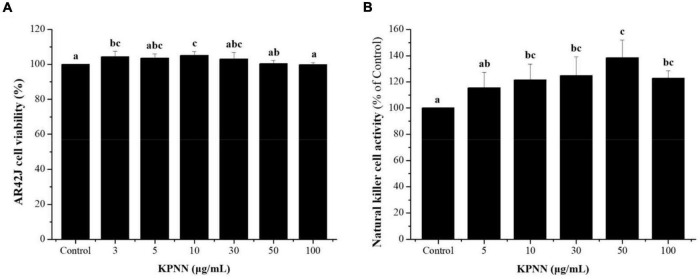
Effects of KPNN on NK cell activity in the splenocytes. **(A)** Splenocytes were co-cultured with target cells (AR42J) in 96-well plates, followed by treatment with KPNN (0, 3, 5, 10, 30, 50, and 100 μg/ml), and incubated for 24 h in a 5% CO_2_ incubator with an effector to target cell ratio of 20:1. **(B)** NK cell activity was calculated as the survival rate of AR42J cells compared to that of the control. Bars labeled with different superscripts indicate significantly different values (*p* < 0.05 vs. control). Data are presented as mean ± SEM (*n* = 3).

An NK cell activity assay was used to evaluate the effect of functional foods on non-specific cell-mediated immunity. Therefore, we examined the effect of KPNN on NK cell activity. Splenocyte cytotoxicity was tested in NK-sensitive tumor cells (AR42J cells). NK cell activity increased after treatment with various concentrations of KPNN (Figure 4A). As shown in Figure 4B, the activity of NK cells significantly increased after exposure to KPNN compared to that of the control group.

### Effect of KPNN on Cytokine Expression in Cy-Treated Splenocytes

**FIGURE 5:**
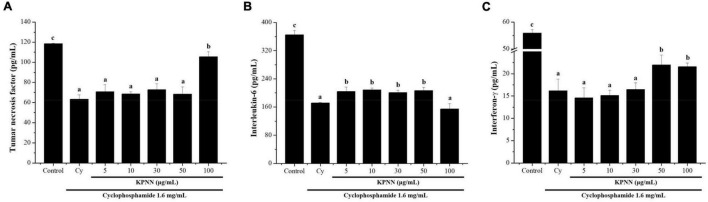
Effects of KPNN on cytokine expression in Cy-treated splenocytes. To analyze the cytokine levels, splenocytes were seeded into a 96-well plate after incubation with Cy (1.6 mg/ml) and KPNN (0, 5, 10, 30, 50, 100 μg/ml) for 24 h in a 5% CO_2_ atmosphere. After 24 h, **(A)** TNF-α, **(B)** IL-6, and **(C)** IFN-γ levels in the culture media were assayed using cytokine activation analysis kits. Data are reported as the mean ± SEM (*n* = 3). *p* < 0.05 vs. control. Bars labeled with different superscript numerals indicate *p* < 0.05.

To determine the effect of KPNN on cytokine production in splenocytes by Cy treatment, the amounts of TNF-α, IL-6, and IFN-γ produced were analyzed. The cells were incubated with Cy (1.6 mg/ml) and KPNN (5, 10, 30, 50, and 100 μg/ml) for 24 h. KPNN increased cytokine levels, confirming that KPNN restored the decreased levels of TNF-α, IL-6, and IFN-γ in Cy-induced immune-reduced splenocytes (Figure 5).

### Effects of KPNN on Thymus and Spleen Damage From Cy-Induced Immunosuppression Rats

**FIGURE 6:**
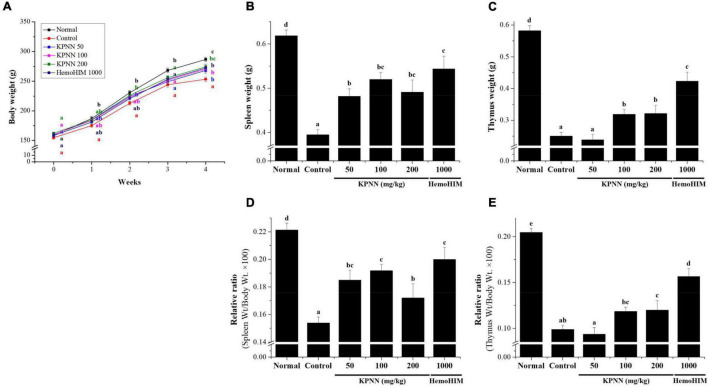
Effects of KPNN on thymus and spleen damage in rats with Cy-induced immunosuppression. The 60 Wistar rats were divided into six groups of 10 animals each and were administered orally for 4 weeks: normal control group (Normal), immunosuppression group (Control), immunosuppression + KPNN 50 mg/kg group (KPNN 50), immunosuppression + KPNN 100 mg/kg group (KPNN 100), immunosuppression + 200 mg/kg group (KPNN 200), and immunosuppression + HemoHIM 1000 mg/kg group (HemoHIM 1000). **(A)** Body weights were measured once per week. The indices of **(B)** spleen and **(C)** relative ratio (weight/body weight × 100), **(D)** thymus, and **(E)** relative ratio (wt./body wt. × 100). Data are expressed as mean ± SEM of three independent experiments, *p* < 0.05 compared to the control group (*n* = 10). Bars labeled with different superscript numerals indicate *p* < 0.05.

To evaluate the effect of a 4-week regimen of KPNN feeding *in vivo*, 6-week-old Wistar rats were orally administered KPNN at three different concentrations (50, 100, or 200 mg/kg). Weekly changes in body weight, water, and food intake were monitored for animals and compared between the groups. The results confirmed that the body weight of the control group administered only Cy (5 mg/kg) was significantly lower than that of the normal control group. In contrast, the group administered various KPNN concentrations in the Cy-induced immunosuppression rat model showed significantly increased body weight compared to that of the control group. The rats in the KPNN 200 and HemoHIM (1,000 mg/kg, positive control) groups registered similar body weights (Figure 6A). To investigate the effect of KPNN on immune-related tissues in an immunosuppressed rat model, the weights of the spleen and thymus tissues were measured. The indices of representative immune organs, i.e., the spleen and thymus, were significantly reduced by oral administration of Cy (Figures 6B,C). However, these decreases in Cy-treated rats were significantly recovered by orally administering KPNN. These findings were similar in terms of relative ratio to body weight (Figures 6D,E).

### Effects of KPNN on Immune Cell Number in Cy-Induced Immunosuppressed Rats

**FIGURE 7:**
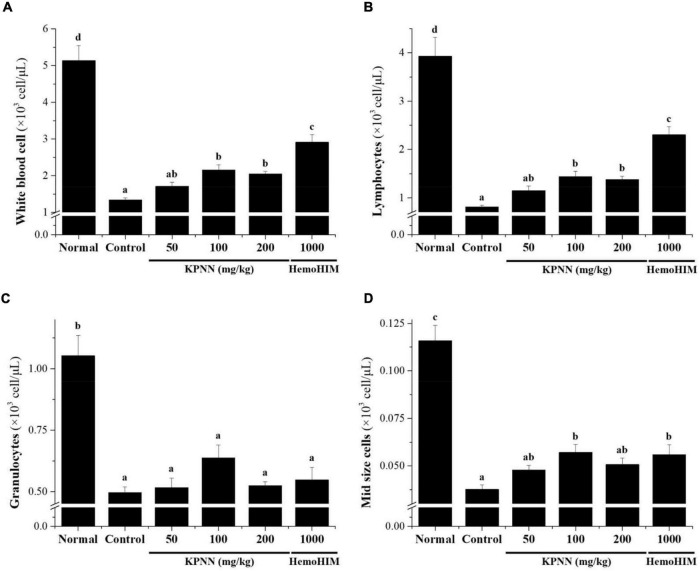
Effects of KPNN on immune cell numbers in Cy. Wistar rats were treated with saline, cyclophosphamide (Cy; 5 mg/kg/day), oral KPNN (0, 50, 100, and 200 mg/kg/day), or oral HemoHIM (1,000 mg/kg/day) once daily for 4 weeks. Whole-blood samples were collected for analysis. The levels of **(A)** total WBCs, **(B)** lymphocytes, **(C)** granulocytes, and **(D)** mid-size cells in the blood samples were determined using a Hemavet 950 system. All data are expressed as mean ± SEM of three independent experiments, *p* < 0.05 vs. control group (*n* = 10). Bars labeled with different superscript numerals indicate *p* < 0.05.

In this study, hematological analysis was performed to confirm the effect of KPNN on the blood immune cell content in immunocompromised rat models. The decreased number of immune cells, such as total white blood cells (WBCs), lymphocytes, mid-size cells (Mid), and granulocytes, in the Cy-induced immunosuppression model were increased in the KPNN-administered group (Figure 7).

### Effect of KPNN on the Cytokine Level of Serum in Cy-Induced Immunosuppressed Rats

**FIGURE 8:**
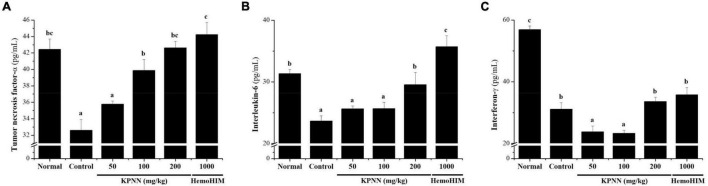
Effect of KPNN on serum cytokine levels in rats with Cy-induced immunosuppression. Wistar rats were treated with saline, cyclophosphamide (Cy; 5 mg/kg/day), oral KPNN (0, 50, 100, and 200 mg/kg/day), or oral HemoHIM (1,000 mg/kg/day) once daily for 4 weeks. For cytokine analysis, blood collected in a conical tube was coagulated at room temperature for 30 min and then separated in a centrifuge at 3000 rpm for 10 min to collect serum. The separated serum was analyzed for **(A)** TNF-α, **(B)** IL-6, and **(C)** IFN-γ levels using an ELISA kit. The bar placed below the graph indicates significant differences at *p* < 0.05 (*n* = 10). Bars labeled with different superscript numerals indicate *p* < 0.05.

To confirm the immune-enhancing effect of KPNN, the serum cytokine content of each group was analyzed 4 weeks after sample administration. In the Cy-treated group, the immune-related cytokines TNF-α, IL-6, and IFN-γ were significantly reduced compared with those in the normal group. In contrast, decreased TNF-α levels in the control group were increased when KPNN ≥ 100 mg/kg was administered, and IL-6 levels were increased when KPNN 200 mg/kg was administered (Figures 8A,B). However, the IFN-γ levels were not significantly different from that in the control group at varying KPNN concentrations (Figure 8C).

### Effects of KPNN on Spleen Damage in Cy-Induced Immunosuppressed Rats

**FIGURE 9:**
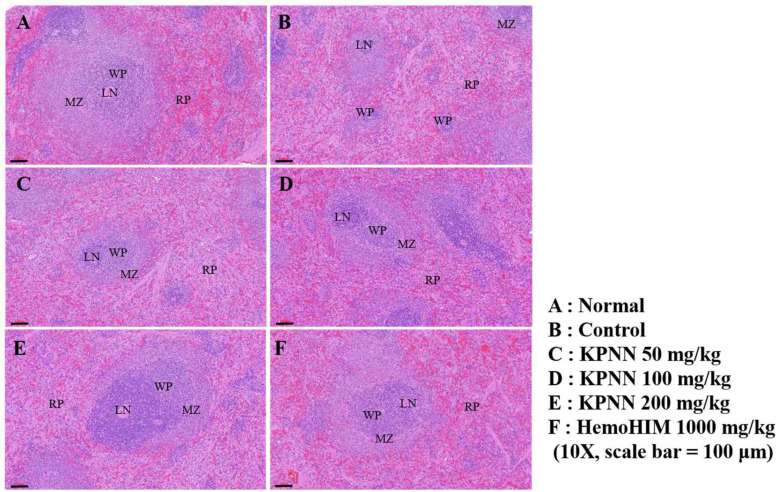
Effects of KPNN on spleen damage in immunosuppressed rats. Wistar rats were treated with saline, cyclophosphamide (Cy; 5 mg/kg/day), oral KPNN (0, 50, 100, and 200 mg/kg/day), or oral HemoHIM (1,000 mg/kg/day) once daily for 4 weeks. Subsequently, damage to the spleen was analyzed histologically. Representative images of sectioned **(A)** normal (saline-treated), **(B)** control (only Cy-treated), and **(C–E)** KPNN-treated rats [treated with Cy and **(C)** KPNN 50 mg/kg, **(D)** KPNN 100 mg/kg, **(E)** KPNN 200 mg/kg, or **(F)** HemoHIM 1000 (HemoHIM 1,000 mg/kg)]. Scale bar = 100 μm. CV, central vein; LN, lymph nodule; MZ, marginal zone; RP, red pulp; WP, white pulp.

We observed the splenic tissue lesions in each group under a microscope to determine the effect of KPNN on morphological changes in the Cy-treated spleens. The white pulp surrounding the central artery in the normal group and the lymph nodes at the edges were visibly demarcated from the red pulp (Figure 9A). However, atrophy of the white pulp and lymphoid depletion was observed in the control group (treated only with Cy), confirming Cy-induced immune suppression (Figure 9B). In the case of the KPNN-administered group, the marginal zone (MZ) region that separates the red and white pulp was not apparent in the KPNN 50 mg/kg group, but white pulp atrophy was better than that of the control group (Figure 9C). KPNN 100 mg/kg group had less white pulp atrophy and relatively minor damage (Figure 9D). This pattern was prominent in the KPNN 200 mg/kg group, where the marginal area around the red pulp was clearly visible, and there was no disruption of the white pulp (Figure 9E). In the positive control group, tissue condensation did not appear, and it was significantly improved compared to that in the control group (Figure 9F). These results showed that KPNN stimulated innate and adaptive immunity by promoting immune-related cytokine production and improving the histopathological characteristics of Cy-induced spleen damage.

## Discussion

Immunity plays a vital role in maintaining homeostasis by inducing biological responses to effectively block external invasions (2, 3). Recently, research has been actively conducted to identify bioactive substances that can alleviate adverse effects on the immune function caused by various drugs used for anti-cancer treatment (1, 8, 25). Although KP or NN extracts as candidate substances are being studied extensively *in vitro* and *in vivo* (13, 15, 17, 19), the effect of KP and NN combination treatment on the immune response was not explored. Therefore, in this study, the immune-enhancing effects of KPNN were investigated using an immunosuppressed animal model. Our results suggest that KPNN extract promoted RAW 264.7 cell proliferation up to a concentration of 100 μg/ml and increased the levels of phospho-NF-κB and phospho-ERK proteins. Furthermore, the activity of NK cells was significantly increased after KPNN exposure. KPNN treatment protected splenocytes from Cy-induced cytotoxicity and increased the levels of immune-related serum cytokines, TNF-α, IL-6, and IFN-γ. Administration of KPNN extract to a Cy-treated immunosuppressed rat model significantly increased body weight compared to that in the control group. The damages in the representative immune organs, such as the spleen and thymus, were significantly alleviated. Furthermore, oral administration of KPNN increased total WBCs, lymphocyte, Mid, and granulocyte counts, as well as the immunity-related serum cytokines, TNF-α and IL-6, and restored normal splenic histology. These results suggest that KPNN restores the immunosuppressive response induced by Cy.

Cy, an alkylating agent with anti-cancer and immunosuppressive properties, has been widely used for immunosuppression in leukemia, rheumatoid arthritis, lymphoma, multiple myeloma, and bone marrow transplantation. However, the non-selective toxicity of Cy renders it toxic to normal cells, thereby causing bone marrow failure, which exacerbates anemia symptoms along with thrombocytopenia, and physiological phenomena, such as poor body growth and decreased immune function (26, 27). Thus, there is an increasing demand to identify and develop bioactive substances capable of lowering the side effects and toxicity of immunosuppressive drugs with non-selective toxicity (28–30).

Splenocytes consist of various cell types with different immune functions, including macrophages, dendritic cells, and T- and B-lymphocytes (31). Macrophages play important roles in both innate and adaptive immune responses. LPS-induced activation of macrophages produces pro-inflammatory cytokines (IL-1, TNF-α, IL-6, and IFN-γ), leading to the activation of phospholipase A2, which produces lipid metabolites of arachidonic acid, such as prostaglandins, and NO. Macrophages also induce MAPK-dependent phosphorylation, thereby activating multiple transcription factors (32–35). LPS upregulates TNF-α and iNOS expression by activating multiple transcription factors and inducing MAPK-dependent phosphorylation. NF-κB is also a major activator of TNF-α production by macrophages (35). Stimulation of splenocyte viability may consequently increase the secretion of cytokines, potentially explaining the observed immune-enhancing and anti-cancer capacity (36). In this study, KPNN-treated RAW 264.7 cells showed increased cell viability in the range of 30–100 μg/ml KPNN concentration compared to that in the control group; however, the NO levels were not significantly altered. Despite the unaltered NO production, these findings suggest that KPNN promotes non-toxic and immune-enhancing effects in macrophages. In contrast, p-ERK and p-NF-κB protein levels in the KPNN-treated group were higher than those in the control and positive control groups. NF-κB and MAPK signaling play essential roles in immune responses (37, 38). A previous study reported that Red *Platycodon grandiflorus* root extract could enhance immunity by increasing the NF-κB phosphorylation level as well as the p38 MAPK-induced NF-κB activation in RAW 264.7 cells (39). KP or herbal mixture including KP showed anti-inflammatory effects by down-regulating IκBα, NF-κB, and JNK/p38 MAP kinase signaling pathways (13, 14, 40–43). In addition, NN exerts anti-inflammatory effects on LPS-stimulated RAW 264.7 macrophages through inhibition of NF-κB and MAPK pathways (44–48). Similar to a previous study (35, 40–48), our results showed that KPNN increased the p-NF-κB and p-ERK in RAW 264.7 cells. These findings are thought to be because of the positive role of KPNN in activating cytokines in Th1 cells, stimulating immune factors in the body, and increasing the activity of NK cells (49). However, downstream signal pathway of these proteins and further studies are required to identify the precise immunomodulatory mechanisms. Collectively, these results suggest that KPNN could induce immunostimulatory effects by regulating ERK and NF-κB signaling in macrophages.

In previous studies, Cy-induced immunosuppression in splenocytes reduced cell proliferation and cytokine levels and suppressed splenic NK cell activity (29, 50). NK cells can target and kill foreign and abnormal cells and play an important role in the early immune response. It is also activated by cytokine and chemokine stimulation and plays a pivotal role in tumor growth, metastasis regulation, and virus clearance (51). Therefore, NK cell activity is a valuable parameter to evaluate the cellular immune response of the host (52). As in previous reports, our results showed that the KPNN-treated group promoted the activity of NK cells and increased the production of cytokines, TNF-α, IL-6, and IFN-γ, in Cy-induced immunosuppressed splenocytes. NK cells were activated by IFN- or macrophage-derived cytokines. Only the infected cells selectively induced apoptosis by recognizing changes in MHC class expression and blocking the activation of uninfected cells (53). KPNN might play an effective role in activating NK cells and increasing cytokine production to remove cells infected by viruses and bacteria. These results suggest that KPNN modulates NK cell activity to improve cell-mediated immune responses.

Cy administration disrupts immune homeostasis in the body by damaging the spleen and thymus, which are representative immune organs that elicit immune responses (54). *In vitro* and *in vivo*, Cy-induced immunosuppression significantly reduced blood cell counts, suppressed the activity of splenic NK cells and Tc cells, and decreased cytokine (IL-2, IFN, and IL-10) levels (55, 56). In this study, as in a previous report (57), the spleen and thymus weights were reduced by Cy treatment, and the decreased indices were recovered upon KPNN treatment. Notably, in the high-dose KPNN group, body weight and spleen weight were restored to that in the HemoHIM (positive control) group. Blood, one of the most important indicators of immune function, defends the body in various ways, such as clotting during bleeding and phagocytosis during bacterial invasion (58). Additionally, immune cells, such as T and B lymphocytes, monocytes, and macrophages, play an important role in regulating the immune response (59). The contents of total leukocytes, lymphocytes, granulocytes, and intermediate cells in the control group were significantly reduced in a Cy dose-dependent manner compared to that in the normal group. In this study, as in a previous report (29), KPNN administration increased the number of total leukocytes, lymphocytes, and mid-size cells, compared to that in the control group. KPNN extract could act on blood cells that perform immune functions in the body, thereby reducing the Cy toxicity and exhibiting an immune-enhancing effect. Collectively, these findings suggest that KPNN enhances immunostimulatory activity by protecting against Cy-induced immune cell damage.

Cytokines perform crucial functions, including lymphocyte differentiation, inflammation regulation, cell survival, apoptosis, and immune responses (60). Among the diverse immune cells, T lymphocytes are essential regulatory cells in the adaptive immune system (61). The different cytokines secreted by T cell subtypes, Th1 and Th2, are important determinants of cell function. Th1 and Th2 cells are mainly involved in cell-mediated and humoral responses, respectively, and promote the secretion of IL-2, TNF-α, and IFN-γ, and IL-4, IL-6, and IL-10, respectively (11, 12, 35). TNF-α is produced by T cells, B cells, NK cells, and macrophages and regulates inflammation and host defense by inhibiting bacterial infection and acute stress (62). IFN-γ promotes Th1 cell differentiation and stimulates B cells to promote Ig production, leading to antibody immunoreactivity (35). Our study on the mouse RAW 264.7 macrophages suggested that KPNN might be involved in the production of macrophage-associated cytokines (TNF-α, IFN-γ, IL-1β, IL-6, and IL-12) *via* MAPK and NF-κB signaling. TLR4 is one of the most widely studied receptors for immune activity, mainly recognizing LPS, lipoic acid, and polysaccharides (35, 63). CLM (*ceriporia lacerata* mycelia) plays an immunostimulatory role in macrophages *via* TLR4-induced TNF-α, IL-1β, and IL-6 production (35). In previous reports, KP treatment inhibited LPS- or TNBS-induced TNF-α and IL-6 expression and production *in vitro* and *in vivo* (40, 42, 43). *In vitro* and *in vivo* studies revealed that NN reduced inflammation through downregulated proinflammatory cytokines TNF-α, IL-6, IL-1β, and IFN-γ in LPS- and DSS-induced inflammation model (44–46). Our results showed that KPNN restored the decreased levels of TNF-α, IL-6, and IFN-γ in a Cy-induced immunosuppressed splenocyte model. Additionally, the reduced TNF-α and IL-6 levels in the Cy-induced immunosuppression rat model were ameliorated by KPNN. However, further studies are required to precisely determine the immune-enhancing effects of KPNN. Collectively, these results suggest that KPNN may play a role in enhancing humoral and cell-mediated immune responses.

The spleen tissue lesions observed in the Cy-treated groups, the collapse of white pulp, and cell coagulation of red pulp observed in the control group tended to improve gradually in the KPNN-treated group. Notably, in the high-concentration KPNN-administered group, the white pulp was evenly distributed around the central vein, and the borders of the margins were visibly separated. These observations suggested that the damage to spleen tissue caused by the immunosuppressed substances was significantly reduced by KPNN treatment. Therefore, KPNN treatment can restore Cy-induced atrophy in the spleen.

## Conclusion

In conclusion, combined treatment with KP and NN extracts increased cell viability, the levels of phospho-NF-κB and phospho-ERK proteins in macrophages, NK cell activity, and cytokine production in splenocytes. *In vivo* studies, KPNN extract was shown to strengthen immunity by increasing body weight, tissue weight, immune cells, and the cytokine content in the blood and reducing Cy-induced damage to the spleen. Therefore, these findings suggest that KPNN treatment effectively enhances immunity and may help develop therapeutic strategies or functional products.

## Data Availability Statement

The original contributions presented in the study are included in the article/Supplementary Material, further inquiries can be directed to the corresponding author.

## Ethics Statement

The animal study was reviewed and approved by the Institutional Animal Care and Use Committee of INVIVO Co., Ltd.

## Author Contributions

YP, HL, DS, DK, JY, HY, MK, and JB: conceptualization and validation. YP, HL, DS, DK, JY, and JB: methodology and formal analysis. YP, DS, DK, JY, and JB: investigation and visualization. YP, HY, MK, and JB: resources and data curation. JB: writing – original draft and reviewing and editing. All authors contributed to the article and approved the final manuscript.

## Conflict of Interest

YP, HL, and DS were employed by INVIVO Co., Ltd. DK and JY were employed by Hanpoong Pharm & Foods Co., Ltd. The remaining authors declare that the research was conducted in the absence of any commercial or financial relationships that could be construed as a potential conflict of interest.

## Publisher’s Note

All claims expressed in this article are solely those of the authors and do not necessarily represent those of their affiliated organizations, or those of the publisher, the editors and the reviewers. Any product that may be evaluated in this article, or claim that may be made by its manufacturer, is not guaranteed or endorsed by the publisher.
