# Supplementary material for: Immune-Enhancing Effects of Co-treatment With Kalopanax pictus Nakai Bark and Nelumbo nucifera Gaertner Leaf Extract in a Cyclophosphamide-Induced Immunosuppressed Rat Model
Source: Front Nutr. 2022 May 19;9:898417. doi: 10.3389/fnut.2022.898417 (PMC9161550; doi:10.3389/fnut.2022.898417)
Supplement: Supplementary file 1 [file Data_Sheet_1.PDF]

Supplementary Material

1 Supplementary Figures and Tables

1.1 Supplementary Figures

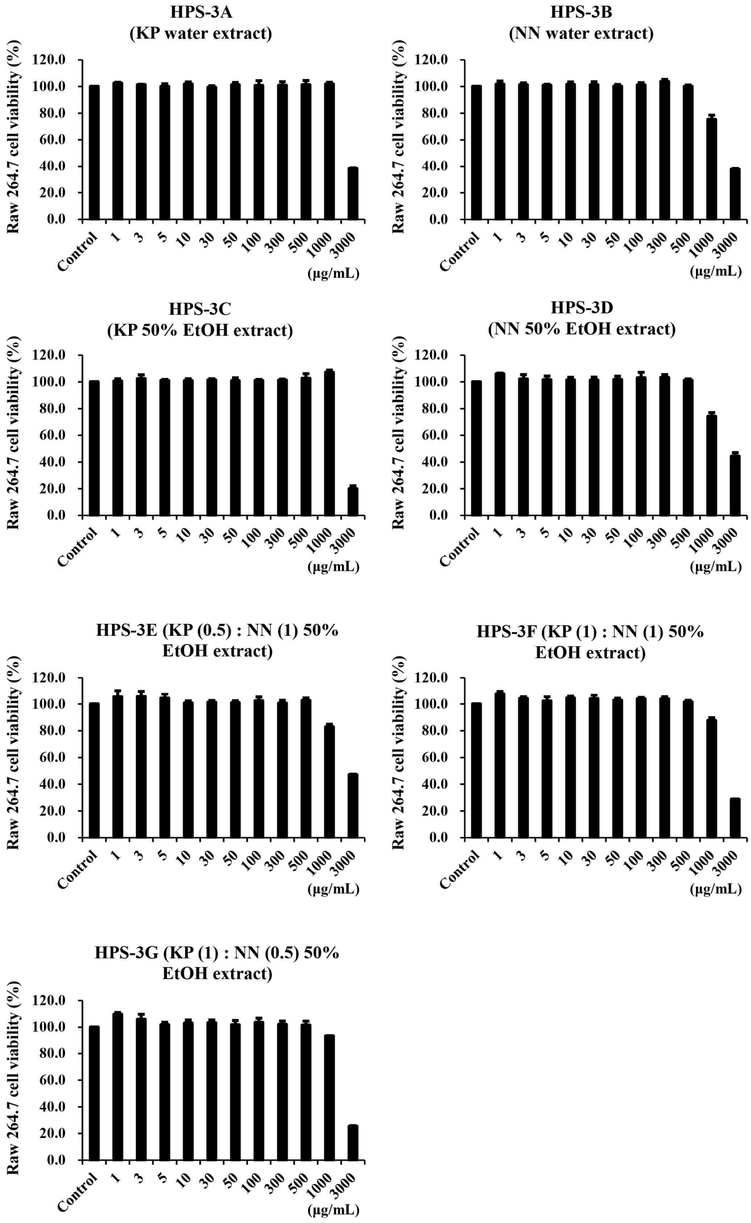

Supplementary Figure 1. Effect of KP, NN and KPNN extracts on cell viability in RAW 264.7 cells. KP and NN sample was extracted in water and 50% ethanol, respectively; HPS-3A: KP water extract, HPS-3C: KP ethanol extract, HPS-3B: NN water extract, HPS-3D: NN ethanol extract. The KPNN sample was extracted by mixing KP and NN in 50% ethanol at a ratio of 0.5:1 (KP:NN, HPS-

3E), 1:1 (HPS-3F), 1:0.5 (HPS-3G). Cells were seeded in 96-well plates (1 x 10^4 cells/90 µl/well) and then KP (HPS-3A, HPS-3C), NN (HPS-3B, HPS-3D) and KPNN (HPS-3E, HPS-3F, HPS-3G) were treated at 0, 1, 3, 5, 10, 30, 50, 100, 300, 500, 1000, and 3000 µg/ml, respectively, and incubated at 37°C and 5% CO_2 for 24 h. Next, cell viability was determined using the WST-1 assay. RAW 264.7 macrophage cytotoxicity was not affected at sample concentrations ≤500 µg/ml and no significant difference was observed up to a concentration of ≤500 µg/ml. Results are presented as mean ± SEM of at least 3 independent experiments (n = 3).

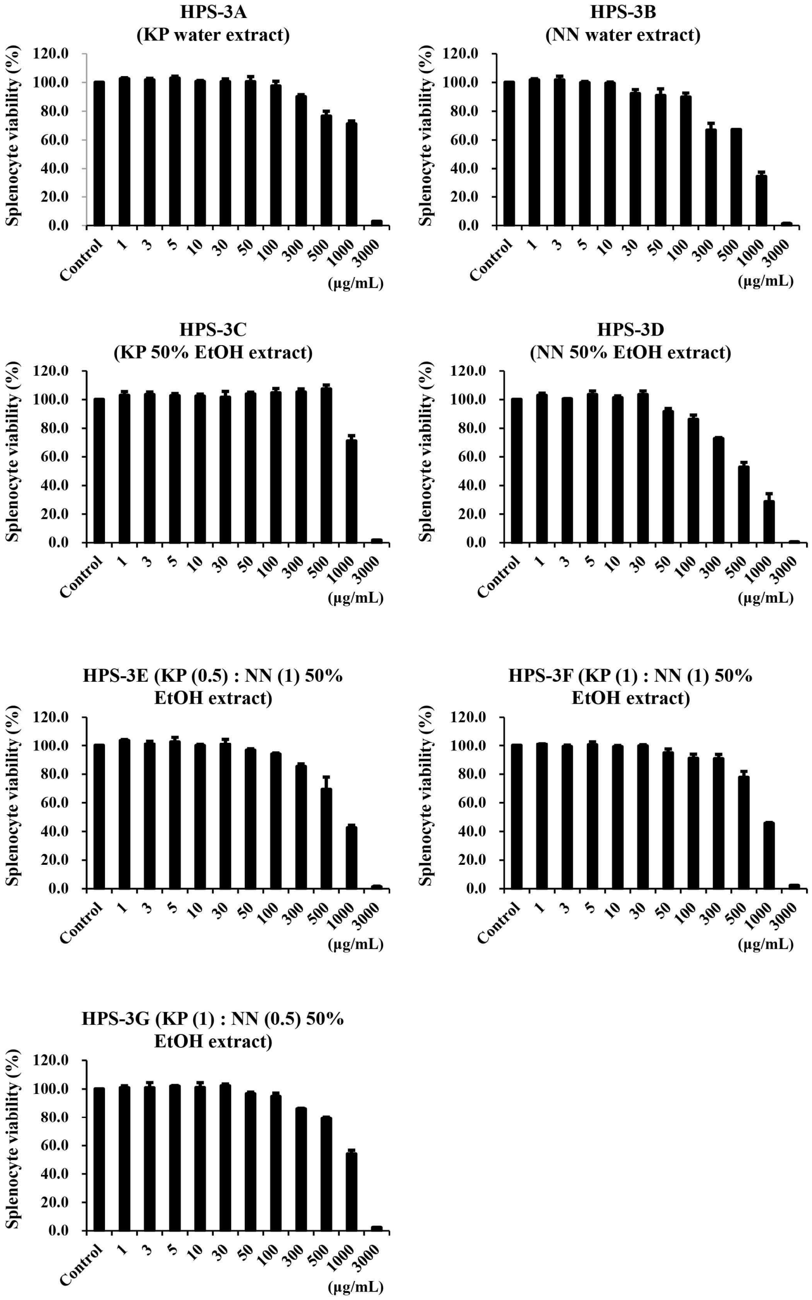

Supplementary Figure 2. Effect of KP, NN and KPNN extracts on cell viability in splenocytes. Isolated splenocytes were seeded in 96-well plates (1 x 10^6 cells/90 µl/well) and then KP (HPS-3A, HPS-3C), NN (HPS-3B, HPS-3D) and KPNN (HPS-3E, HPS-3F, HPS-3G) were treated at 0, 1, 3, 5, 10, 30, 50, 100, 300, 500, 1000, and 3000 µg/ml, respectively. After 24 h, cell viability was measured using the WST-1 assay. Splenocytes cytotoxicity was not affected at sample concentrations ≤100 µg/ml and no significant difference was observed up to a concentration of ≤100 µg/ml. Results are presented as mean ± SEM of at least 3 independent experiments (n = 3).

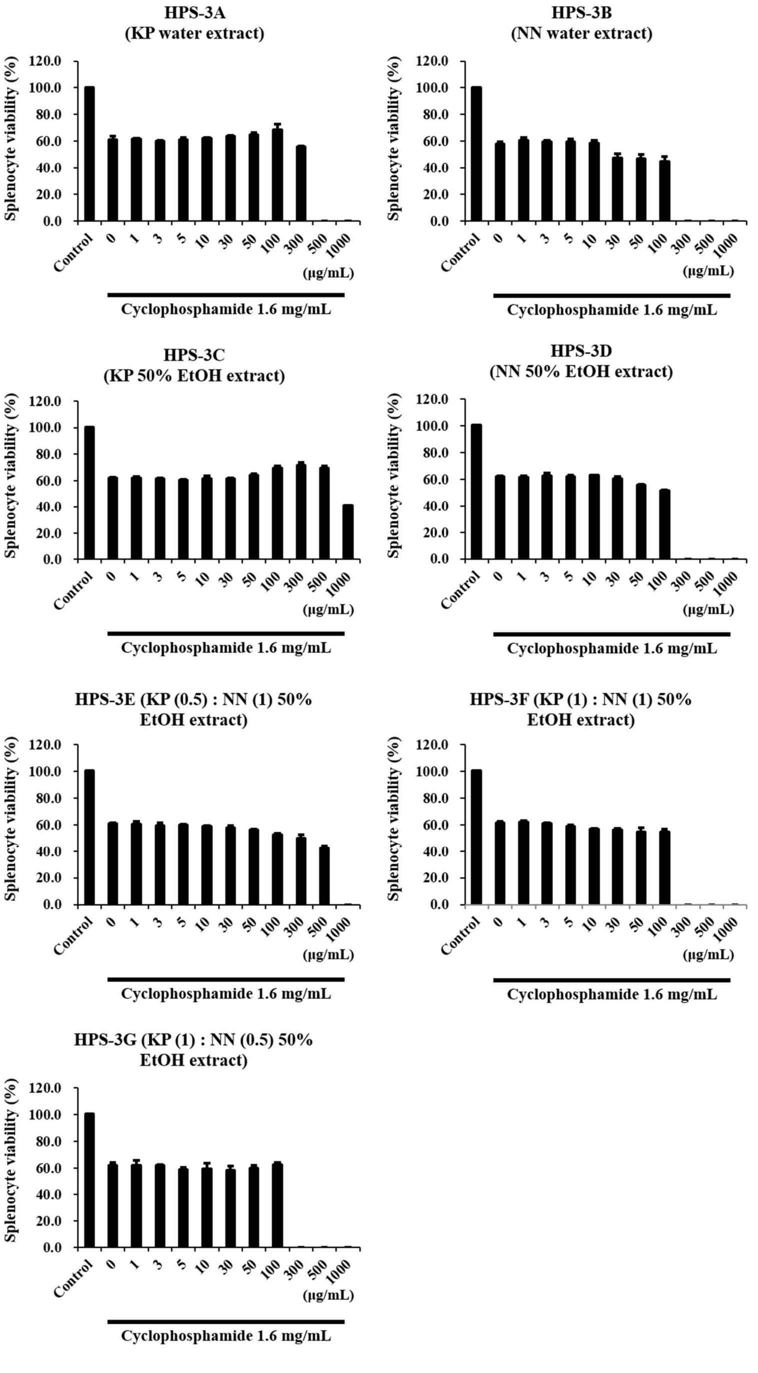

Supplementary Figure 3. Effect of KP, NN and KPNN extracts on cell viability in Cy-induced splenocytes. Isolated splenocytes were seeded in 96-well plates (1 x 10^6 cells/90 µl/well) and then KP (HPS-3A, HPS-3C), NN (HPS-3B, HPS-3D) and KPNN (HPS-3E, HPS-3F, HPS-3G) were treated at 0, 1, 3, 5, 10, 30, 50, 100, 300, 500, and 1000 µg/ml and Cy (1.6 mg/ml), respectively for 24 h. Cell viability was determined using the WST-1 assay. Splenocytes cytotoxicity was not affected at sample concentrations ≤100 µg/ml and no significant difference was observed up to a concentration of ≤100 µg/ml. Therefore, cell viability of Cy-induced splenocytes experiments were performed at a concentration of ≤100 µg/ml. HPS-3G (KP:NN = 1:0.5) sample showed the highest cell viability by Cy treatment, and HPS-3G samples were selected for this study. Results are presented as mean ± SEM of at least 3 independent experiments (n = 3).
